# Editorial: Release of inorganic phosphate from the myosin active site in actomyosin energy transduction

**DOI:** 10.3389/fphys.2026.1823925

**Published:** 2026-03-19

**Authors:** Alf Månsson, Christina Karatzaferi

**Affiliations:** 1 Department of Chemistry and Biomedical Sciences, Faculty of Health and Life Sciences, Linnaeus University, Kalmar, Sweden; 2 Experimental Physiology and Myology Lab-Centre for the Evaluation of Human Performance (CREHP), Department of Physical Education and Sport Science (DPESS), School of P.E., Sport Science and Dietetics, University of Thessaly, Trikala, Greece

**Keywords:** energy transduction, molecular mechanics, muscle mechanics, myosin, muscle fatigue, orthophosphate, power stroke

## Introduction

Eukaryotic cells such as muscle fibres use actin and myosin II to develop force and motion powered by ATP turnover. Reversible release of the ATP hydrolysis product, inorganic phosphate (Pi) from the myosin active site is central in energy transduction and coupled to the force-generating structural change, the power-stroke. The Pi accumulation under conditions of intense physical exercise contributes to muscle fatigue with reversibly reduced force production due to depletion of force-producing actomyosin cross-bridges by Pi-rebinding (Karatzaferi et al., 2004, 2008; Debold, 2012). Such effects also contribute in pathologies with Pi-accumulation in cardiac ischemia (Wu et al., 2008) and premature and persistent muscle fatigue of end-stage renal patients (Johansen et al., 2005). Typically, Pi-release and force generation are both modulated by myosin active-drugs (Trivedi et al., 2020; Spudich, 2024; Miller et al., 2025) developed to target heart and skeletal muscle but also diseases involving non-muscle cells.

Whereas close association of Pi-release and the power-stroke is accepted, the exact timing of involved steps is not. In this topic, three papers review different models (Kaya, Debold et al., Caremani et al.) from early ideas of Pi-release simultaneously with the power-stroke to two-step and multi-step (Caremani et al.) models. The reviews particularly emphasize conflicting evidence from ultrastructural studies, on the one hand, and biophysical functional studies on the other, where the former favour Pi-release before the power-stroke (“Pi-release first”) but some of the latter suggest the opposite (“power-stroke first”) (however, see (Hwang et al., 2021)). The models come in different versions (**Table 1**). “Pi-release first” models generally assume that Pi leaves the active site and reaches solution in a single step. However, recent evidence (Llinas et al., 2015; Moretto et al., 2022) suggest that Pi pauses at secondary sites on myosin on the way from the active site to bulk solution.

**Table 1:** Conceptually different models with different versions for the coupling between Pi-release and power-stroke.

Major model	Main model characteristics	Version of model
1. Pi-release first	Pi-release is assumed to be necessary for the power-stroke to occur (cf (Llinas et al., 2015))	a) Single-step Pi-release (e.g (Smith, 2014))
		b) Multi-step Pi-release (e.g (Moretto et al., 2022))
2. Power-stroke first	Pi-release occurs after the force-generating power-stroke (e.g (Dantzig et al., 1992))	a) Pi-rebinding e.g. in muscle fatigue leads to reversal of the power-stroke (Dantzig et al., 1992)
		b) Pi-rebinding leads to detachment into a post-power-stroke Pi-bound state (e.g (Marang et al., 2025)
3. Loose coupling	Each of several power-stroke transitions into different “mechanical states” are governed by strain in the myosin elasticity, independent of whether Pi and ADP or only ADP is at the active site. Pi-release can occur from any mechanical state but varying conformations lead to different kinetics.	Two elements that seem to be integral parts of the model (but that may possibly be omitted) are myosin slippage transitions between actin sites and a branched pathway with irreversible detachment (with certain probability) into a pre-power-stroke Pi-bound state

In “power-stroke first” models, Pi-rebinding is generally believed to reverse the power-stroke. However, alternatively [e.g (Marang et al., 2025)] Pi-rebinding may guide detachment of myosin from actin into states outside the conventional actomyosin cycle. Ideas that Pi-release and the power stroke are loosely coupled, with Pi-release from any structural and mechanical state (details in **Table 1**), are also considered particularly in Caremani et al., elaborating on a comprehensive model (Caremani et al., 2013). Another consideration (Caremani et al.) is a higher ([Pi]-sensitivity of force than ATP turnover rate in isometrically contracting fast skeletal muscle. The proposed branched kinetic scheme is challenged by Månsson suggesting that the effect is instead explained by different kinetics of myosin cross-bridges attaching to actin under variable elastic strain.

Also in this topic, original work by Stehle, demonstrates a negative, non-linear correlation between the effects of varied [Pi] on force and the rate of isometric force development in cardiac myofibrils. Tests of the results against six single-pathway models of the cross-bridge cycle, suggest either slow Pi binding or slow force reversal. However, the models do not consider strain-dependence of transition rates which might alter the conclusions [cf. (Månsson, 2025) and (Månsson)]. Nonetheless, the non-linear negative correlation described by Stehle adds to the list of phenomena to explain.

Li et al. used quantum dots (QDs), whose fluorescence is reduced upon increased local temperature, to study critical illness myopathy [CIM; (Friedrich et al., 2018)] in a rat intensive care model. They show that myosin extracted from muscle fibre bundles rich in type I myosin, from CIM animals, exhibited universal increase in heat production consistent with decrease in myosin efficiency. Beyond insights into the CIM pathogenesis, novel methodology is presented for analyzing free energy changes during actomyosin cycling dominated by Pi-release from myosin.

Finally, a theoretical paper (Månsson) considers the mechanism of action of a small molecule myosin-activating compound, omecamtiv mecarbil, highlighting the central role of Pi-release in the mechanism of myosin-active drugs.

## Perspectives and conclusions

Different papers in this topic present intricate experimental and modelling data to support specific models for the coupling between Pi-release and force-generation (**Table 1**). However, conclusive evidence is lacking. Limitations include different assumptions used in data interpretation and absence of methodological means for direct observation of critical details. As pointed out by Debold et al., a key goal would be to visualize the location of Pi on myosin while simultaneously detecting the lever arm position. A recent time-resolved cryo-EM study (Klebl et al., 2025) may be a step towards that goal. Another issue which complicates data interpretation is the difficulty of calibrating all model parameter values against experimental results (Månsson, Månsson) to create coherence between work on isolated proteins and large myosin ensembles interacting with actin filaments, outside or inside an organized sarcomere. Moreover, macro-level experiments, e.g. from single muscle fibres, are affected by biomechanical complexities, e.g. sarcomere non-uniformities [cf (Stehle, 2017; Rassier and Månsson, 2025)], not considered in modelling. Furthermore, from a physiological point of view, accumulation of Pi occurs together with proton accumulation and temperatures above 30 °C (for mammals), with myosin types presenting differing kinetics changes under fatigue (Karatzaferi et al., 2017). This emphasizes the importance of assessing the effects of varied [Pi] while considering modulating and synergistic roles of other factors, particularly protons and temperature but also ADP and myosin light chain phosphorylation (Karatzaferi et al., 2004, 2008; Debold, 2012).

Beyond uncertainties regarding whether Pi-release occurs before or after the power stroke or if there is loose coupling between power stroke and Pi-release, there are other outstanding issues. This includes the possible role of Pi-binding on myosin outside the active site and what happens upon Pi rebinding to myosin. Moreover, the possibility of different mechanisms in different myosin isoforms was brought up (e.g. in Kaya, Stehle) as well as the need to obtain more experimental data under coherent conditions to calibrate model parameter values in a consistent way. Finally, modelling using simple kinetic schemes *vs*. more complex mechanokinetic models requires further consideration.

To conclude, the present Research Topic gives an insightful overview of current understanding and suggests interesting paths forward.
